# GP’s perspectives on laboratory test use for monitoring long-term conditions: an audit of current testing practice

**DOI:** 10.1186/s12875-020-01331-6

**Published:** 2020-12-05

**Authors:** Martha M. C. Elwenspoek, Ed Mann, Katharine Alsop, Hannah Clark, Rita Patel, Jessica C. Watson, Penny Whiting

**Affiliations:** 1 The National Institute for Health Research Applied Research Collaboration West (NIHR ARC West), University Hospitals Bristol NHS Foundation Trust, 9th Floor, Whitefriars, Lewins Mead, Bristol, BS1 2NT UK; 2 Population Health Sciences, Bristol Medical School, University of Bristol, Bristol, BS8 2PS UK; 3 Tyntesfield Medical Group, Bristol, BS48 2XX UK; 4 Nightingale Valley Practice, Bristol, BS4 4HU UK; 5 Brisdoc Healthcare Services, Bristol, BS14 0BB UK

**Keywords:** General practice, Optimal testing, Chronic disease monitoring

## Abstract

**Background:**

We have shown previously that current recommendations in UK guidelines for monitoring long-term conditions are largely based on expert opinion. Due to a lack of robust evidence on optimal monitoring strategies and testing intervals, the guidelines are unclear and incomplete. This uncertainty may underly variation in testing that has been observed across the UK between GP practices and regions.

**Methods:**

Our objective was to audit current testing practices of GPs in the UK; in particular, perspectives on laboratory tests for monitoring long-term conditions, the workload, and how confident GPs are in ordering and interpreting these tests. We designed an online survey consisting of multiple-choice and open-ended questions that was promoted on social media and in newsletters targeting GPs practicing in UK. The survey was live between October–November 2019. The results were analysed using a mixed-methods approach.

**Results:**

The survey was completed by 550 GPs, of whom 69% had more than 10 years of experience. The majority spent more than 30 min per day on testing (78%), but only half of the respondents felt confident in dealing with abnormal results (53%). There was a high level of disagreement for whether liver function tests and full blood counts should be done ‘routinely’, ‘sometimes’, or ‘never’ in patients with a certain long-term condition.

The free text comments revealed three common themes: (1) pressures that promote over-testing, i.e. guidelines or protocols, workload from secondary care, fear of missing something, patient expectations; (2) negative consequences of over-testing, i.e. increased workload and patient harm; and (3) uncertainties due to lack of evidence and unclear guidelines.

**Conclusion:**

These results confirm the variation that has been observed in test ordering data. The results also show that most GPs spent a significant part of their day ordering and interpreting monitoring tests. The lack of confidence in knowing how to act on abnormal test results underlines the urgent need for robust evidence on optimal testing and the development of clear and unambiguous testing recommendations. Uncertainties surrounding optimal testing has resulted in an over-use of tests, which leads to a waste of resources, increased GP workload and potential patient harm.

**Supplementary Information:**

The online version contains supplementary material available at 10.1186/s12875-020-01331-6.

## Background

The number of tests ordered by GPs has tripled over the last 20 years [1]. One of the reasons for this increase is a growing ageing population with complex health needs. In addition, over the last 20 years, many secondary care services have been diverted to primary care [2]. The Quality and Outcomes Framework, which was introduced on 1 April 2004, incentivises laboratory testing for long-term conditions and has been associated with an increase in GP consultation rates [3]. Around half of the diagnostic tests ordered in primary care are for monitoring long-term conditions [4]. However, routine testing is supported by limited evidence and much of it may be unnecessary.

The over-use of tests has contributed to increased NHS spending and an ever-rising GP workload. UK primary care spends an estimated £1.8bn per year on laboratory tests [1]. The total costs are probably higher, because this figure does not account for the cost of GPs time reviewing test results or the administration of processing testing data. Time spent on reviewing test results was estimated at 25–35 min per day for GPs in 2000/1 and increased to 1.5–2 h per day in 2015/16 [1]. Typical working days for GPs are currently between 10 and 14 h long [5]. Over-testing also increases the risk of false positive test results, which can cause anxiety for patients and unnecessary additional GP appointments, phlebotomy or medical imaging (i.e. ultrasound, X-ray, CT scan, MRI) appointments, and referrals – risking avoidable harm to patients [6–8].

To reduce healthcare costs, efforts have been made to identify unwarranted variation in testing. Geographical variation in testing is unwarranted when it cannot be explained by variation in population needs. There is substantial variation in test ordering across the UK between GP practices and regions [9]. A recent systematic review of UK guidelines shows that current recommendations for monitoring hypertension, type 2 diabetes, and chronic kidney disease (CKD) are largely based on expert opinion [10]. Due to a lack of robust research evidence on optimal monitoring strategies and testing intervals, the guidelines are unclear and incomplete [10]. This uncertainty may underlie unwarranted variation in testing.

To audit current testing practices of GPs in the UK, we developed a questionnaire-based survey that was sent out to GPs across the UK. The main focus of the audit was on GP perspectives on laboratory tests in monitoring long-term conditions, workload involved in testing, and how confident GPs are in ordering and interpreting tests results.

## Methods

### Study design

The survey was developed by academics and GPs with a special interest in optimal testing for long-term conditions in primary care using OnlineSurveys, an online survey tool designed for academic research. OnlineSurveys use an ISO 27001 certified information security management system [11] and comply with the General Data Protection Regulation [12]. Survey questions were piloted in the GP practices of the co-authors and amongst GPs of the Optimal Testing Group, a subgroup of the Royal College of General Practitioners (RCGP) Overdiagnosis group [13].

### Survey dissemination

The survey was available from any computer or mobile device at any time between 7 October and 30 November 2019. The survey was endorsed by the RCGP who helped promote the survey in their newsletter and clinical news article. In addition, the survey was disseminated via the national GP research network practices (PCRN), doctors.net forum, and social media. All GP practising in the UK were eligible to fill out the survey.

### Survey questions

The survey consisted of 14 multiple-choice questions and one free-text question. Most multiple-choice questions included an ‘other’ option, which individuals could specify. Three questions were on demographics (sex, location, years since qualification). Nine questions covered workload, confidence in underlying evidence and how to deal with abnormal test results, perspectives on the number of tests being done, importance of research in optimal testing, and risk of patients harm due to over-testing, processes that determine which tests are being done, and how often tests are done for secondary care. The final multiple-choice question was optional and asked which tests the GPs perform routinely, sometimes, or never, for an average adult patient with hypertension, type 2 diabetes, or CKD. At the end of the survey, individuals had the opportunity to add any comments or questions about testing in primary care. See Additional file 1 for the full questionnaire.

### Data analysis

We used descriptive statistics (percentages, medians, inter quartile ranges (IQR)) to describe the quantitative findings of the survey. The UK has four increasingly distinct healthcare systems in England, Wales, Northern Ireland, and Scotland; therefore, we performed a sensitivity analysis by limiting the analysis to GPs practicing in England. The analysis was performed in Stata 15.1 [14].

As Question 17 (“which tests do you perform routinely, sometimes, or never, for an average adult patient with hypertension, type 2 diabetes, or CKD”) was optional, we only included respondents in the analysis who fully answered this question. Respondents were asked to select one of three options (*routinely*, *sometimes*, or *never)* per test and condition. If respondents selected more than one option, only the higher frequency option was included in the analysis (i.e. if both “never” and “sometimes” were selected, it was counted as “sometimes”). However, if both “never” and “routinely” were selected for the same test in the same condition, the answer was deemed invalid and excluded from analysis. We performed an exploratory analysis to identify significant relationships between the level of testing and explanatory variables (i.e. sex, region, workload, level of experience or confidence) using Chi-squared tests. Levels of testing were categorised as ‘low’, ‘medium’, and ‘high’ according to GPs self-reported testing behaviour for all three conditions.

To identify patterns and themes within the answers given to the final free text question, we used an inductive thematic analysis as described by Braun and Clarke [15], which is a bottom-up approach to explore qualitative data. KA and ME coded the free-text answers and analysed the responses by identifying higher-level recurring themes and looked for suggested solutions.

## Results

### Sample demographics

A total of 550 individuals completed the survey (Table 1). Fifty per cent of respondents identified as male. We received responses from all major geographical regions in the UK, although most GPs (*n* = 170) were practicing in the South West of England at the time of completing the survey. The fewest responses were from Northern Ireland (*n* = 3). Most respondents had more than 10 years’ experience as a GP (*n* = 383) (Table 1).

**Table 1:** Sample demographics

	n (%)
**Sex** (male)	279 (50.73)
**Region**	
North West	22 (4.01)
London	44 (8.01)
South West	170 (30.97)
West Midlands	55 (10.02)
South Central	37 (6.74)
South East Coast	18 (3.28)
Scotland	87 (15.85)
East of England	15 (2.73)
Wales	12 (2.19)
Northern Ireland	3 (0.55)
Yorkshire and Humber	19 (3.46)
East Midlands	57 (10.38)
North East	10 (1.82)
**Experience (years)**	
0–5	75 (13.64)
5–10	92 (16.73)
> 10	383 (69.64)

### Tests in primary care: workload, confidence and perspectives

**Fig. 1:**
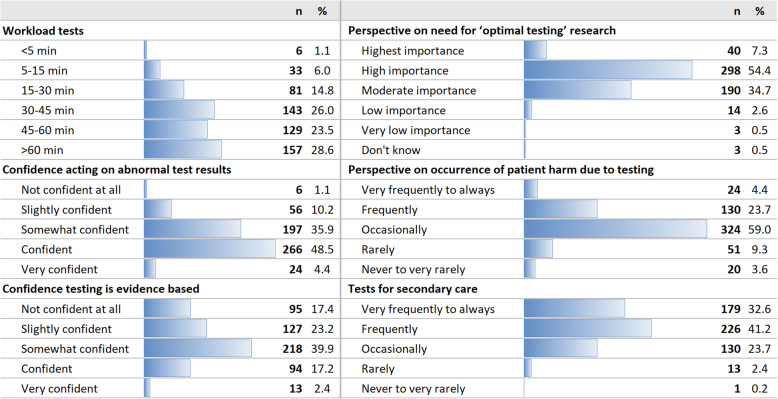
Tests in primary care: workload, confidence and perspectives

On their most recent full practice day, 429 (78%) GPs spent more than 30 min on ordering, interpreting and acting on laboratory tests for routine monitoring of long-term conditions (including time for ordering tests, reviewing test results, talking to patients about the results and any action resulting from the original test, such as further testing or referrals). 157 (28.6%) GPs had spent more than 1 h of this practice day on tests (Fig. 1).

290 (52.8%) GPs felt confident (of which 24 very confident) in what to do with abnormal incidental findings picked up during long-term disease monitoring and 259 (47%) GPs felt less than confident. 440 (80.4%) GPs were less than confident that the tests they perform are evidence-based. 338 (61.7%) GPs believed that further research on optimising testing in primary care is of high importance. 478 (87.1%) GPs believed that blood test monitoring can at least occasionally be harmful to patients. Many GPs are asked to do tests for secondary care outside of a shared care agreement – 405 (73.8%) GPs indicated this happens frequently to very frequently (Fig. 1). Only 343 (62.7%) were able to download secondary care blood results into their clinical system.

GPs indicated that they used one or more of the following processes in their practice for deciding which blood tests to use: a practice protocol (*n* = 354, 64.4%), the quality and outcomes framework (*n* = 254, 46.2%), NICE clinical guidelines (*n* = 175, 31.8%), and laboratory electronic test ordering profiles (e.g. ICE profiles in England) (*n* = 197, 35.8%) 143 (26%) GPs used bespoke plans for individual patients and 32 (5.8%) did not know.

We performed a sensitivity analysis to explore the implications of limiting the analysis to the responses from GPs practicing in England. Excluding responses from Wales (*n* = 12), Scotland (*n* = 89), and Northern Ireland (*n* = 3) had limited effect on the results (changing the responses by no more than 1–2%) and did not affect the overall trends (Additional file 2).

Tests used for patients with hypertension, type 2 diabetes, and chronic kidney disease.

Fifty-six percent of respondents answered the optional question on which tests they ordered routinely, sometimes or never for hypertension, type 2 diabetes, and CKD. The following results are based on their answers only.

**Fig. 2:**
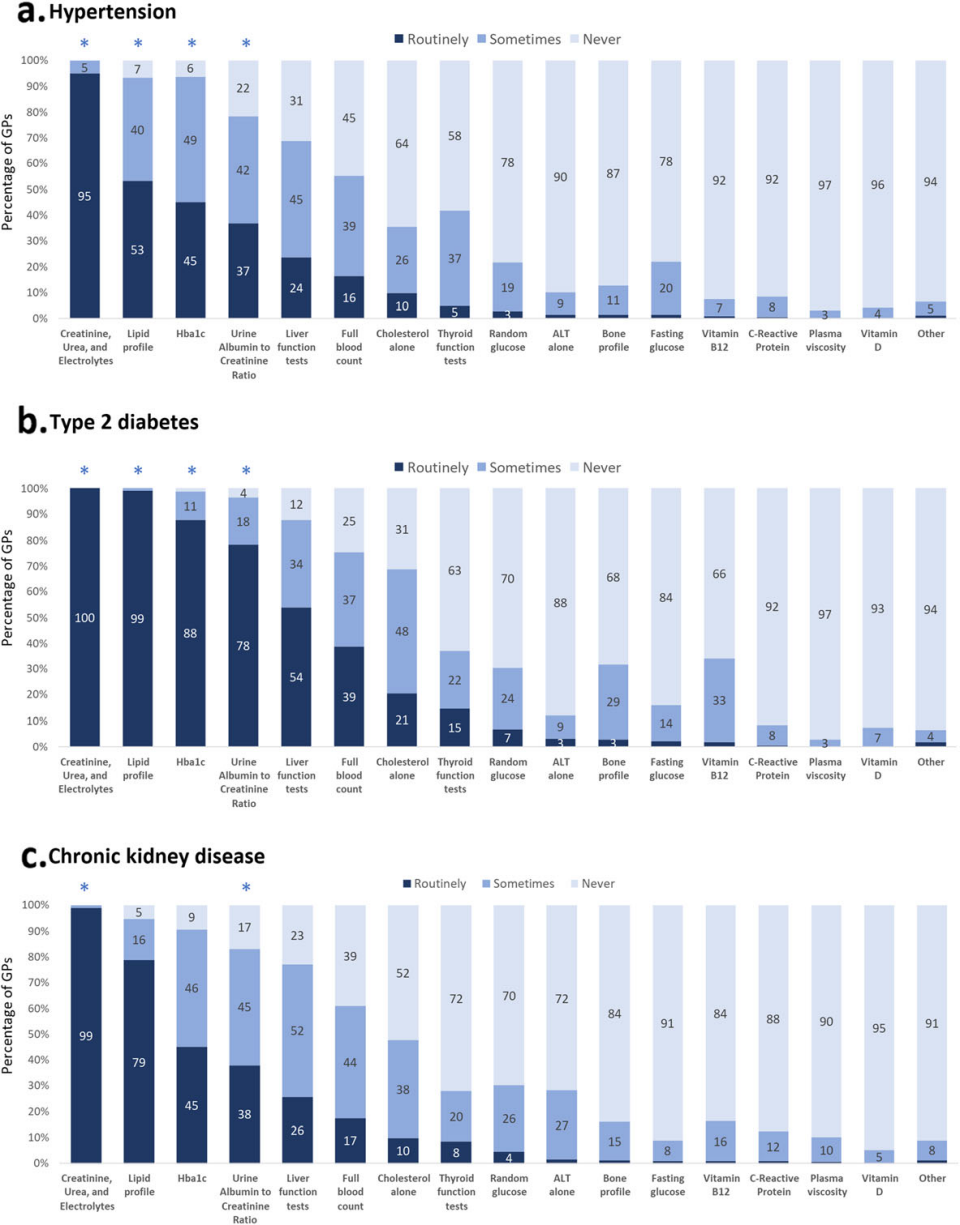
Test ordering for an average adult patient with a common long-term condition. These graphs show the percentage of GPs that prescribe certain tests never, sometimes or routinely to patients with hypertension (**a**), type 2 diabetes (**b**), or CKD (C). The star (*) indicates tests that are recommended by current UK guidelines to be done routinely in these patient groups

For patients with hypertension, type 2 diabetes, and CKD, GPs ordered a median of 3 (IQR 2–4), 5 (IQR 4–6), and 3 (IQR 2–5) tests routinely, respectively. Creatinine, Urea, and Electrolytes was the most common test that was ordered routinely for all three patient groups (> 95%). We found a high level of disagreement (i.e. variation) for a number of tests, where ‘never’, ‘sometimes’, and ‘routinely’ were selected by an almost equal numbers of GPs. This was especially the case for liver function tests (LFTs) and full blood count (FBC) in all three conditions (Fig. 2). We found little evidence of any pattern that could explain this level of disagreement (i.e. sex, region, workload, level of experience or confidence) (Additional file 3).

Current UK guidelines recommend routine testing of creatinine, urea, and electrolytes, lipid profile, HbA1c, and urine albumin to creatinine ratio in patients with hypertension [16–18]. Most GPs (*n* = 288, 95.4%) routinely ordered creatinine, urea, and electrolytes for hypertension; however, only half or less of the GPs routinely ordered lipid profile (*n* = 156, 52.0%), HbA1c (*n* = 121, 39.9%), or urine albumin to creatinine ratio (*n* = 102, 33.7%). Several tests that are not specifically mentioned by current guidelines were done routinely by some GPs, such as FBC (*n* = 58, 19.3%) and LFTs (*n* = 79, 26.1%) (Fig. 2a).

For all patients with diabetes, current UK guidelines recommend routine testing of creatinine, urea, and electrolytes [18, 19], urine albumin to creatinine ratio [18, 19], and HbA1c [20, 21]. These test are performed routinely by most GPs in our study sample: 99.3% (*n* = 301), 87.5% (*n* = 265), and 99.0% (*n* = 300), respectively. Although less clearly mentioned in the NICE and SIGN guidelines [19, 20, 22], the Clinical Knowledge Summaries (CKS) recommend assessing a person’s full lipid profile annually [23], which was performed routinely by 75.2% (*n* = 227) of the GPs. Other tests that GPs selected, which are not mentioned by guidelines, were LFTs (*n* = 173, 57.1%), FBC (*n* = 125, 41.3%), and thyroid function tests (*n* = 69, 22.9%) (Fig. 2b). New thyroid disease guidelines recommend to ‘not offer testing for thyroid dysfunction solely because an adult, child or young person has type 2 diabetes’. [24].

UK guidelines recommend testing creatinine, urea, and electrolytes [18, 21], and urine albumin to creatinine ratio [18] at least annually in patients with CKD. These were done routinely by 99.0% (*n* = 301) and 77.2% (*n* = 233), respectively, of the GPs in our sample. NICE CKD guidelines also recommend to check the haemoglobin level in people with a GFR of less than 45 ml/min/1.73 m^2^ [18], which was done routinely by 47.5% (*n* = 144) of GPs in our sample. A substantial number of GPs also routinely ordered lipid profile (*n* = 114, 37.9%) and HbA1c (*n* = 68, 22.6%) for the average patient with CKD, which are not specifically mentioned by current guidelines (Fig. 2c).

### Common themes in GP comments

Twenty-four per cent of respondents left a comment in the free text box at the end of the survey. We identified three overarching themes and 11 sub-themes in these comments (Table 2), which are discussed below, as well as solutions suggested by respondents.

**Table 2:** Participant themes and sub-themes

Themes	Sub-themes
Pressures that promote over-testing	Quality and Outcomes Framework (QOF)/ guidelines/protocol Workload from secondary care Fear of missing something Patient led demand/expectation Doing more tests at once to save nurse appointments later
Consequences of over-testing	Workload Harms of testing
Uncertainty	Lack of evidence for clinical practice How frequently to monitor Doctors being uncertain about what other staff nurses/healthcare assistants are doing in their treatment room Cost of tests

#### Pressures that promote over-testing

GPs identified several pressures that may cause GPs to order more tests than necessary. Many GPs mentioned guidelines, practice protocols, and the Quality and Outcomes Framework (QOF), that promote extra and possibly unnecessary testing:

“*[I] believe [testing] should be for a particular reason and at a time that is right for the patient and find it frustrating that 'guidelines' and QOF insist on GPs doing lots of unnecessary tests.”*

GPs feel they have no choice but to follow these protocols, even if they believe a test is not necessary and a waste of resources. GPs mentioned pressure from colleagues to adhere to the protocols,

“I am under huge pressure from ‘guideline addicts’ in my practice”

or concerns about medico-legal consequences if they don’t,

“*It does not feel safe from a medico-legal point of view to reduce testing.*”

GPs believe extra tests are added out of fear of missing something and many unnecessary tests are ordered “just in case” or “to be safe”. One GP wrote:

“*I think we work in a defensive way, fearing litigation or complaints if we miss something*.”

GPs are increasingly expected to manage and follow up tests requested by secondary care. One GP reported,

“[A] huge additional workload from secondary care redirecting requests to primary care”.

GPs are concerned about the large volume of duplicate tests and repeat tests in response to slight abnormalities picked up in secondary care, as well as requests to monitor treatments without clear guidance or to follow up on test results while it is unclear why that test was requested in the first place. GPs also mentioned the pressure from patients themselves, who expect and ask for extra tests:

*“[The] main issue is patients requesting tests, not just 'routine' reviews,”* and *“I feel under pressure to request too many [ … ] and am at pains to explain to patients that results may not give the reassurance they seek.”*

Another reason mentioned for doing more tests than necessary is to avoid additional future nurse appointments:

*“When I try to rationalise tests, something else happens to the [patient] and I wish I’d just done the whole raft of tests to save multiple trips for patients and appointments for us.”*

#### Consequences of over-testing

GPs mentioned several negative consequences of over-testing, especially the increased workload:

“*I am overwhelmed with all the routine monitoring and it has risen massively over the 20+ years I have been qualified,”*

which is negatively effecting job satisfaction:

“The monitoring of results is sinking the morale of GPs due to the volume of work.”

A few GPs acknowledged the waste of resources,

“*I regard the over-investigation of essentially well patients on an annual basis as a simply scandalous waste of NHS resource as well as a frequent source of trivial results which require further tests!*”

and the possible harm unnecessary testing has on patients,

“*The harms and distress caused by testing and explaining minor abnormalities is huge.”*

#### Uncertainty

GPs need to deal with many uncertainties, which complicates optimal monitoring for long-term conditions. One major problem is the lack of evidence on how, when, and how often patients should be monitored to improve their outcomes. One GP explained,

*“We have recently updated our long-term condition protocols and it has been difficult to find evidence-based literature on how frequently and what type of blood tests should be ordered routinely for long-term conditions.”*

Some GPs are uncertain about what other staff nurses/health care assistants are doing in their treatment rooms:

“O*ne of the challenges is that bloods are often requested by another member of the [multidisciplinary team] (e.g. [practice nurse] or pharmacist) making it more difficult and time-consuming to interpret, and often creating additional workload.*”

Many GPs are unaware of the costs associated with the tests that they order:

*“We are not given any indication of the costs of tests (despite asking laboratory) to allow cost-effectiveness to be considered.”*

#### Suggested solutions

GPs ask for clear guidelines on the optimal frequency of testing, on when/what not to test to avoid litigation and complaints, and on what to do with slightly abnormal test results. The lack of evidence needs to be addressed and feed into new evidence-based guidelines. Guidelines need to recommend a minimal testing set and specify how to personalise testing strategies. GPs said,

“*It would be nicer to have a more bespoke approach with really good evidence base guidelines to help us*”, and

“*It would be really good to have a MINIMAL set of evidence based routine monitoring for these conditions”*.

Several GPs highlighted the issue of having to review test results that someone else has ordered for unknown reasons, for instance by secondary care doctors, practice nurses, or pharmacists. Some GPs believe that the number of unnecessary test ordering and the workload for GPs to follow up on results will reduce when test results must be checked by the person that ordered them. For example,

“*Secondary care doctors should be bound to check their own results, not tell patients to 'see your GP’,*” and

“*One of the challenges is that bloods are often requested by another member of the [multidisciplinary team] [ … ]. The tests should go back to the requesting clinician where possible.”*

The number of unnecessary tests and follow up testing can be reduced significantly when duplication of tests from secondary care are avoided. This can be achieved by having access to their results and compatible IT systems:

*“Lots of tests might be avoided if it was known that it had been done recently in hospital. Avoiding all this would save hours of GP time, hours of phlebotomy time and £££ in the lab, not to mention appointment staff and clogged up car park,”* and

*“Large problem is being able to access results from secondary care - to avoid duplication.”*

More awareness is needed among GPs and other staff involved in test ordering regarding the costs of tests and the potential patient harm. To achieve this, GPs suggest the following:

*“I think routinely the cost of each test should be next to it… would a GP just click on the ESR* (Educational Supervisor’s Review) *if it was £10 ‘just because’ or would they pause to consider if their differential includes GCA* (giant cell arteritis) *or suspected Rheum disease?”,* and

*“Totally agree that unnecessary tests cause distress and patient anxiety and this needs to be taught at medical school - how not to follow a protocol and use clinical judgement.”*

Finally, one GP suggested that:

“*Patient [s] should be obliged to pay privately for tests that are not necessary according to the GP or guidelines*”.

## Discussion

### Summary

A considerable part of a GPs working day is filled with ordering or reviewing tests. Still, only half of the respondents felt confident in dealing with abnormal results. GPs answers varied substantially on testing frequency for patients with either hypertension, type 2 diabetes, or CKD. This was especially true for LFTs and FBC, where almost equal numbers of GPs indicated they order these tests ‘routinely’ or ‘never’.

Most GPs routinely ordered tests that are recommended by current UK guidelines for patients with hypertension, type 2 diabetes, or CKD. Exceptions were urine albumin to creatinine ratio for CKD and hypertension patients and HbA1c for hypertension patients, which are recommended by guidelines but were ordered ‘never’ or ‘sometimes’ by most GPs. In addition, many GPs perform additional tests on a routine basis that are not recommended by guidelines, such as lipid profile and LFTs, although some of these tests may have been selected because GPs assumed that they need to monitor for medications in these patient groups. Testing outside guidelines is de facto screening.

The free text comments revealed three common themes: (1) pressures that promote over-testing, including guidelines or practice protocols, workload from secondary care, fear of missing something, patient requests and expectations, (2) negative consequences of over-testing, such as increased workload and patient harms, and (3) uncertainties due to lack of evidence and unclear guidelines, which in itself can be a cause of over-testing.

GPs suggested several solutions, such as better alignment with secondary care testing to avoid duplication and increasing awareness on costs and harms of testing. Above all, GPs highlighted the need for clear evidence-based guidelines.

### Strengths and limitations

A strength of this audit is the combination of both quantitative and qualitative data and analysis methods. The open text box allowed GPs to highlight issues in their own words that were particularly important to them. These data provided important context for interpreting the quantitative data. The anonymity of respondents encouraged GPs to answer the questions honestly, especially on questions about confidence and to raise additional issues in the free text space. It was not possible to calculate a response rate to the survey, as we do not know how many GPs were reached through social media dissemination, however the number of responses exceeded our sample size calculation.

Limitations included the representativeness of the sample. The study participants consisted of a self-selected sample and so, GPs interested in this area of research or GPs who felt strongly about issues related to testing were more likely to respond. Therefore, the perspectives of the study sample are not necessarily representative of all GPs in the UK. Although we received responses from all areas of the UK and equal numbers of responses from men and women, there was an overrepresentation of GPs practicing in the South-West area, where our team is based. There was a lack newly qualified GPs among the survey participants, which may have led to an underestimation of uncertainty and variation in testing.

Our audit targeted GPs only, although nurses, healthcare assistants, and pharmacists also play a role in test ordering. The number of non-GP clinicians in primary care is rising [25] and some evidence suggests that this is linked to an increased use of tests [26]. Thus, a major limitation of this study is that this group of individuals was not included.

### Comparison with existing literature

Our findings chime with previous work that showed large geographical variation in test ordering behaviour among GPs [1, 9]. These studies used routinely collected primary care data, which do not contain data on the GPs reasons for testing. In contrast, our survey highlights which tests GPs order for an average adult patient to monitor a specific long-term condition. To our knowledge, this is the first study that shows the variation in GP test ordering behaviour for the same fictional ‘average adult patient’.

GPs selected a number of additional tests that are not recommended by UK guidelines. A review of testing panels used for monitoring long-term conditions among 20 GP practices in North Devon found that no two practices recommended the same set of tests [27]. We suspect that much of the additional testing is the result of local practice protocols. Most practices in North Devon included regular LFTs and FBC in their chronic disease monitoring protocols [27]. We found large variation in the use of these two tests in our study sample. Our results suggest that routine testing for LFT and FBC occurs UK-wide, despite the fact that these tests are not recommended by national guidelines.

### Implications for research and practice

Although guidelines lack a strong evidence base, they represent best current practice. However, current guidelines are often unclear about frequency of testing and testing recommendation for long-term conditions are spread over several different documents [10]. Because there are no clear national testing protocols to follow, GP practices develop and use their own. New primary care-based guidelines are needed that specifically inform the use of tests in monitoring long-term conditions. In absence of clear evidence, the use of minimal testing sets with longer testing intervals should be encouraged. Efforts should be made to align local practice protocols with these new testing guidelines. Reducing unnecessary testing has been suggested as an important priority to help ensure value-based primary care [28]. In North Devon, a quality improvement project that set out to ‘standardise the blood tests used for monitoring of chronic conditions in primary care’ resulted in a 14% reduction for FBC testing and a 22% reduction for LFTs, without reducing the number of tests showing possible significant pathology [27]. Our survey and the North Devon review suggest that aligning local practice protocols can reduce unnecessary testing without a reduction in detection rate for significant abnormality. These guidelines should also protect GPs from complaints and litigation when patients demand additional (and potentially unnecessary) tests.

Increasing awareness of costs related to testing among GPs, practice nurses, and healthcare assistants will enable them to incorporate costs into their decision-making. In addition, primary care clinicians need to be aware of patient harms caused by over-testing. The risk of false positive results is high in a low prevalence setting such as primary care, especially for tests that have been ordered without clear indication or rationale. GPs should consider offering training to nursing staff or healthcare assistants who are involved in test ordering about the potential harms of over-testing. Openly sharing uncertainty and fallibility regarding diagnostic tests with patients and colleagues has been suggested as a promising strategy to prevent the harm of over-testing [29]. Although shared-decision making is widely accepted as best practice, most research has focused on treatment decisions rather than investigations. Sharing uncertainty to help facilitate shared decision making can be challenging in clinical practice – patient and doctors have been shown to have limited understanding of health statistics and risk [30], and tend to overestimate the benefits of tests [31].

GPs should be able to access test results from secondary care to avoid duplication. Only 63% of GPs in our sample was able to do this. This should be a priority for the NHS because precious resources are being wasted due to inefficient communication and incompatible IT systems between primary and secondary care.

## Conclusion

In order to improve guidelines, we need strong evidence on what testing strategies inform patient care and improve patient outcomes. One of the main problems with current guidelines is that they promote more care (prescribe more, intervene more, refer more) rather than less [32, 33]. Although implementation of evidence-based guidelines can improve patient care and outcomes [34], they do not reduce the workload of the clinicians. Also, there is a reluctance to take out recommendations of existing guidelines as long as they do not cause harm, even if new evidence suggest there is no clear benefit [34]. To reduce stress and workload, we need to identify routine services that can be stopped or scaled back [35]. Future research should provide evidence on how to safely reduce the frequency of testing, what to do with slightly abnormal test results (and associate risks of patient outcomes) and identify minimal testing sets.

## Supplementary Information

**Additional file 1.** Survey questions.

**Additional file 2.** Sensitivity analysis limited to responses from GPs practicing in England. Tests in primary care: workload, confidence and perspectives.

**Additional file 3.** Relationships between test prescribing and explanatory variables.

## Data Availability

The datasets generated during and/or analysed during the current study are available from the corresponding author on reasonable request.

## Abbreviations

ALT: Alanine aminotransferase
CKD: Chronic Kidney Disease
CKS: Clinical Knowledge Summaries
FBC: Full Blood Count
GFR: Glomerular Filtration Rate
GP: General Practitioner
HbA1c: Hemoglobin A1c
ICE: Integrated Clinical Environment
IQR: Interquartile Range
LFT: Liver Function Test
NHS: National Health Service
NICE: National Institute for Clinical Excellence
PCRN: Primary Care Research Network
QOF: Quality Outcomes Framework
RCGP: Royal College of General Practitioners
SIGN: Scottish Intercollegiate Guidelines Network.
