# Supplementary material for: GP’s perspectives on laboratory test use for monitoring long-term conditions: an audit of current testing practice
Source: BMC Fam Pract. 2020 Dec 5;21:257. doi: 10.1186/s12875-020-01331-6 (PMC7719260; doi:10.1186/s12875-020-01331-6)
Supplement: Supplementary file 1 — Additional file 1. Survey questions. [file 12875_2020_1331_MOESM1_ESM.pdf]

Optimising testing

Blood tests in primary care - have your say

We are a group of GPs trying to explore ways to optimise chronic disease monitoring in primary care with the aim of reducing GP workload and improving patient care. We are supported by the Royal College of General Practitioners and researchers from NIHR ARC West and the University of Bristol.

By filling out this survey you will help us understand your perspectives on laboratory tests in chronic disease monitoring, the workload and how confident you are in ordering and interpreting these tests.

This survey will take about 5 minutes to complete.

Contact: Martha Elwenspoek, NIHR ARC West, martha.elwenspoek@bristol.ac.uk

NIHR ARC West = National Institute for Health Research Applied Research Collaboration West (previously NIHR CLAHRC West)

Screening

1. Do you work as a GP or GP trainee in primary care in the UK?

- ○ Yes
- ○ No

About you

2. What is your gender?

- Male
- Female

3. Please select the region of your GP practice.

4. Years since qualification as a GP:

- 0-5
- 6-10
- More than 10
- GP trainee

Tests in primary care

5. On your most recent full practice day, how much time did you spend on ordering, interpreting and acting on laboratory tests for routine monitoring of chronic conditions? Include time for ordering tests, reviewing test results, talking to patients about the results and any action resulting from the original test (e.g. further testing or referrals)?

- <5 min
- 5-15 min
- 15-30 min
- 30-45 min
- 45-60 min
- >60 min

6. How confident are you that the tests you do for chronic disease monitoring are evidence based?

- Not confident at all
- Slightly confident
- Somewhat confident
- Confident
- Very confident

7. How confident are you in what to do with abnormal incidental findings picked up during chronic disease monitoring?

- Not confident at all
- Slightly confident
- Somewhat confident
- Confident
- Very confident

8. What is your personal feeling on how many tests are done for chronic disease monitoring?

Please don't select more than 1 answer(s) per row.

	Too few	Slightly too few	About right	Slightly too many	Too many	Not sure
Hypertension	☐	☐	☐	☐	☐	☐
Type 2 Diabetes	☐	☐	☐	☐	☐	☐
Chronic kidney disease	☐	☐	☐	☐	☐	☐

9. How important do you think 'optimising testing in primary care for chronic disease monitoring' is as a topic for further research?

- ○ Highest importance
- ○ High importance
- ○ Moderate importance
- ○ Low importance
- ○ Very low importance
- ○ Absolutely no importance
- ○ Don't know

10. Do you believe blood test monitoring in chronic diseases can be harmful for patients?

- ○ Always
- ○ Very Frequently
- ○ Frequently
- ○ Occasionally
- ○ Rarely
- ○ Very Rarely
- ○ Never

11. What process(es) is/are there in your practice for deciding which blood tests are done for chronic disease monitoring?

- ☐ A practice protocol

- ☐ Using the laboratory electronic test ordering profiles (e.g. ICE profiles)
- ☐ Bespoke for individual patients
- ☐ Clinical Commissioning Group protocol
- ☐ NICE Clinical Guidance
- ☐ Quality and Outcomes Framework (QOF)
- ☐ I don't know
- ☐ Other

11.a. If you selected Other, please specify:

12. Are you asked to do tests for secondary care (outside of a shared care agreement)?

- ○ Always
- ○ Very Frequently
- ○ Frequently
- ○ Occasionally
- ○ Rarely
- ○ Very Rarely
- ○ Never

13. Are you able to download secondary care blood results into your clinical system?

- ○ Yes
- ○ No

Phlebotomy

14. Do you have on site phlebotomy?

- Yes
- No

14.a. If you don't have on site phlebotomy, how far do patients have to travel? Please answer in minutes to travel in normal conditions.

Please enter a whole number (integer).

15. How many collections do you have per day?

- 1
- 2
- 3
- 4
- Other

15.a. If you selected Other, please specify:

16. Do you have ability to centrifuge?

- Yes
- No

OPTIONAL ADDITIONAL QUESTION

We would like to understand more about what tests you order for patients with hypertension, type 2 diabetes and CKD. This information would be extremely helpful for us, but we appreciate that this question may take a little more time to answer than previous questions. Therefore, this question is optional.

17. What tests do you do *routinely* or *sometimes* order for the following conditions (in the average adult patient)?

	Hypertension			Type 2 Diabetes			Chronic kidney disease (CKD3 or above)		
	routinely	sometimes	never	routinely	sometimes	never	routinely	sometimes	never
Full blood count	☐	☐	☐	☐	☐	☐	☐	☐	☐
Creatinine, Urea, and Electrolytes	☐	☐	☐	☐	☐	☐	☐	☐	☐
Liver function tests	☐	☐	☐	☐	☐	☐	☐	☐	☐
ALT alone	☐	☐	☐	☐	☐	☐	☐	☐	☐
Lipid profile	☐	☐	☐	☐	☐	☐	☐	☐	☐
Cholesterol alone	☐	☐	☐	☐	☐	☐	☐	☐	☐
Hba1c	☐	☐	☐	☐	☐	☐	☐	☐	☐
Fasting glucose	☐	☐	☐	☐	☐	☐	☐	☐	☐
Random glucose	☐	☐	☐	☐	☐	☐	☐	☐	☐
Thyroid function tests	☐	☐	☐	☐	☐	☐	☐	☐	☐
Urine Albumin to Creatinine Ratio	☐	☐	☐	☐	☐	☐	☐	☐	☐

C-Reactive Protein	☐	☐	☐	☐	☐	☐	☐	☐	☐
Plasma viscosity	☐	☐	☐	☐	☐	☐	☐	☐	☐
Vitamin B12	☐	☐	☐	☐	☐	☐	☐	☐	☐
Vitamin D	☐	☐	☐	☐	☐	☐	☐	☐	☐
Bone profile	☐	☐	☐	☐	☐	☐	☐	☐	☐
Other	☐	☐	☐	☐	☐	☐	☐	☐	☐

17.a. If you have ticked anything in the other box - please state what test, why you order this and in which group of patients.

Comments

18. If you have any additional comments or questions about testing in primary care, please feel free to write them here.

Thank you for participating

Please contact Martha Elwenspoek, NIHR CLAHRC West (martha.elwenspoek@bristol.ac.uk) if you have any queries.

Key for selection options

3 - Please select the region of your GP practice.

North West
London
South West
West Midlands
South Central
South East Coast
Scotland
East of England
Wales
Northern Ireland
Yorkshire and Humber
East Midlands
North East
