# Supplementary material for: GP’s perspectives on laboratory test use for monitoring long-term conditions: an audit of current testing practice
Source: BMC Fam Pract. 2020 Dec 5;21:257. doi: 10.1186/s12875-020-01331-6 (PMC7719260; doi:10.1186/s12875-020-01331-6)
Supplement: Supplementary file 2 — Additional file 2. Sensitivity analysis limited to responses from GPs practicing in England. Tests in primary care: workload, confidence and perspectives. [file 12875_2020_1331_MOESM2_ESM.pdf]

Additional file 2

Sensitivity analysis limited to responses from GPs practicing in England. Tests in primary care: workload, confidence and perspectives.

	n	%		n	%
**Workload tests**			**Perspective on need for 'optimal testing' research**		
<5 min	**5**	1.1	Highest importance	**33**	7.4
5-15 min	**28**	6.3	High importance	**252**	56.5
15-30 min	**62**	13.9	Moderate importance	**143**	32.1
30-45 min	**126**	28.2	Low importance	**12**	2.7
45-60 min	**101**	22.6	Very low importance	**3**	0.7
>60 min	**125**	28.0			
**Confidence acting on abnormal test results**			**Perspective on occurrence of patient harm due to testing**		
Not confident at all	**5**	1.1	Very frequently to always	**17**	3.8
Slightly confident	**46**	10.3	Frequently	**108**	24.2
Somewhat confident	**149**	33.3	Occasionally	**260**	58.2
Confident	**225**	50.3	Rarely	**45**	10.1
Very confident	**22**	4.9	Very rarely to never	**17**	3.8
**Confidence testing is evidence based**			**Tests for secondary care**		
Not confident at all	**84**	18.8	Very frequently to always	**144**	32.2
Slightly confident	**97**	21.8	Frequently	**183**	40.9
Somewhat confident	**170**	38.1	Occasionally	**109**	24.4
Confident	**84**	18.8	Rarely	**11**	2.5
Very confident	**11**	2.5	Very rarely to never	**0**	0.0
