# Supplementary material for: GP’s perspectives on laboratory test use for monitoring long-term conditions: an audit of current testing practice
Source: BMC Fam Pract. 2020 Dec 5;21:257. doi: 10.1186/s12875-020-01331-6 (PMC7719260; doi:10.1186/s12875-020-01331-6)
Supplement: Supplementary file 3 — Additional file 3. Relationships between test prescribing and explanatory variables. [file 12875_2020_1331_MOESM3_ESM.pdf]

Additional file 3

Relationships between test prescribing and explanatory variables.

		Level of testing			Chi^2 test
		**Low**	**Medium**	**High**	**P value**
**Sex (n, %)**	Male	53 (54.1)	47 (44.8)	50 (49.5)	0.414
	Female	45 (45.9)	58 (55.2)	51 (50.5)	
**Region (n, %)**	North West	4 (4.1)	2 (1.9)	4 (4.0)	0.606
	London	4 (4.1)	4 (3.8)	11 (11.0)	
	South West	35 (35.7)	29 (27.6)	23 (23.0)	
	West Midlands	9 (9.2)	17 (16.2)	11 (11.0)	
	South Central	9 (9.2)	7 (6.7)	8 (8.0)	
	South East Coast	2 (2.0)	2 (1.9)	3 (3.0)	
	Scotland	12 (12.2)	14 (13.3)	17 (17.0)	
	East of England	1 (1.0)	4 (3.8)	6 (6.0)	
	Wales	3 (3.1)	2 (1.9)	2 (2.0)	
	Northern Ireland	1 (1.0)	0 (0)	1 (1.0)	
	Yorkshire and Humber	5 (5.1)	5 (4.8)	3 (3.0)	
	East Midlands	12 (12.2)	18 (17.1)	9 (9.0)	
	North East	1 (1.0)	1 (1.0)	2 (2.0)	
**Year of experience as GP (n, %)**	0 – 5 years	10 (10.2)	12 (11.4)	13 (12.9)	0.974
	6 – 10 years	17 (17.4)	19 (18.1)	16 (15.8)	
	More than 10 years	71 (72.5)	74 (70.5)	72 (71.3)	
**Time spent on ordering and interpreting test results (n, %)**	Less than 30 min/day	28 (28.6)	17 (16.2)	12 (11.9)	0.058
	30 – 45 min/day	23 (23.5)	30 (28.6)	29 (28.7)	
	45 – 60 min/day	24 (24.5)	29 (27.6)	23 (22.8)	
	More than 60 min/day	23 (23.5)	29 (27.6)	37 (36.6)	
**Confidence testing guidelines are evidence based (n, %)**	Not confident at all	23 (23.7)	12 (11.4)	14 (14.1)	0.211
	Slightly confident	23 (23.7)	30 (28.6)	23 (23.2)	
	Somewhat confident	36 (37.1)	43 (41.0)	44 (44.4)	
	Confident	14 (14.4)	20 (19.1)	15 (15.2)	
	Very confident	1 (1.0)	0 (0)	3 (3.0)	
**Confidence acting on abnormal test results (n, %)**	Not confident at all	1 (1.0)	1 (1.0)	2 (2.0)	0.575
	Slightly confident	8 (8.2)	10 (9.5)	6 (5.9)	
	Somewhat confident	28 (28.6)	44 (41.9)	37 (36.6)	
	Confident	56 (57.1)	44 (41.9)	52 (51.5)	
	Very confident	5 (5.1)	6 (5.7)	4 (4.0)	
